# Effects of Malted Rice Amazake on Constipation Symptoms and Gut Microbiota in Children and Adults with Severe Motor and Intellectual Disabilities: A Pilot Study

**DOI:** 10.3390/nu13124466

**Published:** 2021-12-14

**Authors:** Suzumi Kageyama, Rikako Inoue, Koji Hosomi, Jonguk Park, Hitomi Yumioka, Tomo Suka, Yoshihiro Kurohashi, Kazuaki Teramoto, A. Yasmin Syauki, Miki Doi, Haruka Sakaue, Kenji Mizuguchi, Jun Kunisawa, Yasuyuki Irie

**Affiliations:** 1 Graduate School of Health and Welfare Science, Okayama Prefectural University, Okayama 719-1197, Japan; suzumi.kageyama@gmail.com (S.K.); syaukiyasmin@gmail.com (A.Y.S.); doimiki1214@gmail.com (M.D.); skaaue71@gmail.com (H.S.); 2 Department of Nutritional Science, Faculty of Health and Welfare Science, Okayama Prefectural University, Okayama 719-1197, Japan; rinoue@fhw.oka-pu.ac.jp; 3 Laboratory of Vaccine Materials, Center for Vaccine and Adjuvant Research, National Institutes of Biomedical Innovation, Health and Nutrition (NIBIOHN), Osaka 567-0085, Japan; hosomi@nibiohn.go.jp (K.H.); yumioka@osaka-seikei.ac.jp (H.Y.); kunisawa@nibiohn.go.jp (J.K.); 4 Laboratory of Gut Environmental System, National Institutes of Biomedical Innovation, Health and Nutrition (NIBIOHN), Osaka 567-0085, Japan; 5 Artificial Intelligence Center for Health and Biomedical Research, National Institutes of Biomedical Innovation, Health and Nutrition (NIBIOHN), Osaka 567-0085, Japan; jonguk@nibiohn.go.jp (J.P.); kenji@nibiohn.go.jp (K.M.); 6 Faculty of Nutrition, Osaka Seikei College, Osaka 533-0007, Japan; 7 Department of Rehabilitation, Kishu Rehabilitation Visiting Care Station, Wakayama 640-0332, Japan; wmoons116@gmail.com (T.S.); muku-hana.4496@outlook.jp (Y.K.); kisyuuriha9261@wit.ocn.ne.jp (K.T.); 8 Department of Nutrition, Faculty of Medicine, Hasanuddin University, Makassar 90245, Indonesia; 9 Institute for Protein Research, Osaka University, Osaka 565-0871, Japan

**Keywords:** malted rice amazake, severe motor and intellectual disabilities, constipation, constipation assessment scale (CAS), gut microbiota, 16S rRNA

## Abstract

Constipation is a frequent complication in patients with severe motor and intellectual disabilities (SMID). The aim of this study was to investigate changes in constipation symptoms and gut microbiota associated with the intake of malted rice amazake, a fermented food in Japan, in patients with SMID. Ten patients consumed the test food for six weeks, and their physical condition, dietary and medication status, and constipation assessment scale (CAS) were investigated. Comprehensive fecal microbiome analysis using the 16S rRNA sequence method was performed. The results showed a significant decrease in CAS, and a significant increase in Lactobacillales and decrease in *Escherichia-Shigella* after consuming malted rice amazake. To investigate the difference in the effects of malted rice amazake consumption, based on the characteristics of the original gut microbiota, the patients were grouped according to the similarity of their gut microbiota before the intervention; Firmicutes-rich Group 1 (*n* = 5), Actinobacteria-rich Group 2 (*n* = 4), and Proteobacteria-rich Group 3 (*n* = 1). The CAS decreased in Groups 1 and 2. The relative abundance of *Bifidobacterium* showed an increasing tendency both overall and in Group 1, but it was originally higher in Group 2. Our results suggest that malted rice amazake consumption reduces constipation symptoms and simultaneously changes the gut microbiota, but the changes may vary depending on the original composition of the gut microbiota.

## 1. Introduction

Patients with severe motor and intellectual disabilities (SMID) have multiple health problems that require constant medical care. According to a recent systematic review, as many as 35 symptoms, including epilepsy, pulmonary and respiratory diseases, hearing impairment, gastroesophageal reflux disease, and visual impairment, have been reported [1]. Among these symptoms, constipation has been reported to occur in 57% of the children with severe cerebral palsy [2] and 94% of the children with SMID [3], making constipation a frequent complication in patients with SMID.

For patients with SMID, constipation can cause serious problems such as gastroesophageal reflux disease, aspiration and breathing problems, as well as psychological changes, such as pain and behavioral problems, effects on extremity spasticity, and even death from intestinal obstruction [4,5,6]. Causes of constipation include skeletal deformity due to underlying diseases such as cerebral palsy, excessive muscle tone, decreased intestinal peristalsis, resulting in difficulty in defecation, lifestyle factors such as inadequate water intake, lack of fiber in the diet, and lack of exercise, as well as the effects of therapeutic drugs, such as anticonvulsants [2,7,8].

To improve constipation, a multifaceted approach, centered on medication and dietary therapy, in addition to physical therapy such as voiding exercises, is generally used [9]. The management of constipation in patients with SMID is often left to caregivers because it is difficult to determine constipation symptoms from communication, such as complaints and exchange of information related to abdominal symptoms. Laxatives and enemas are mainly used [3,10]. Once laxatives and enemas are used, many patients with SMID will need to continue them throughout their lives, and there are problems such as diarrhea caused by laxatives becoming a factor in urinary tract infections [11], habitual use and tolerance of laxatives and enemas, leading to an increase in the amount and type of drugs, and the use of enemas affecting the perception of bowel movements [8,10]. These factors can cause physical and mental stress in patients with SMID and increase the burden on the family and caregivers [8,10]. Dietary fiber optimization has been reported to be effective in the treatment of chronic constipation in patients whose dietary fiber intake is inadequate [9]. Due to respiratory management, such as tracheostomy and using a ventilator, or due to feeding and swallowing difficulties, the diet of patients with SMID often consists of enteral feeds and pastes with low or no fiber content, which may worsen constipation [2,7,8]. Although dietary therapy is expected to improve constipation because it has fewer side effects than medication, dietary therapy focusing on dietary fiber has limitations in the management of constipation in patients with SMID because of difficulties in feeding and swallowing. Dietary therapy is expected to establish a practical method to improve constipation in clinical practice and at home, considering the characteristics of patients with SMID.

Fermented foods, along with dietary fiber [9], have been reported to improve constipation [8,10,12]. Among Japanese fermented foods, amazake has been traditionally consumed since ancient times, and in recent years, it is often consumed as a nutritious beverage. There are few safety concerns about weight gain and increasing blood sugar levels [13]. Because of its thicker constitution, amazake is easier to ingest than foods containing a lot of dietary fiber, which require chewing and swallowing. Amazake can also be consumed by people who are allergic to milk, which is a concern with products such as fermented milk and lactic acid bacteria beverages. Moreover, unlike amazake made from sake lees, amazake made from malted rice does not contain alcohol, so it can be consumed by people of all ages. There have been several reports on the improvement of constipation due to the consumption of amazake. In a human intervention study on amazake from sake lees, it was reported that the consumption of 750 mg of amazake improved the quality, quantity, and odor of defecation, and it gave a feeling of refreshment after defecation [14]. In an intervention study of minor-grain amazake, for the elderly with constipation, spontaneous defecation was observed with 100 mL of minor-grain amazake, indicating that it is useful for improving constipation [15]. In addition, the effects of malted rice amazake on constipation among female college students [16], as well as middle-aged and older adults [17] have also been reported. These findings indicate that amazake consumption may be effective in improving chronic constipation.

Constipation is closely related to gut microbiota. Hundreds of species of bacteria inhabit the human intestinal tract and interact with the host, either directly or through their metabolites, to influence host nutrition, drug efficacy, physiological function, aging, carcinogenesis, immunity, and infection [18]. Therefore, it is important to understand and control the function of the gut microbiota as an organ composed of heterogeneous organisms for health maintenance and disease prevention [19].

The relationship between gut microbiota and constipation has been reported in various studies on the analysis of gut microbiota, using next-generation sequencing in addition to culture and polymerase chain reaction (PCR) methods, in recent years. In particular, oligosaccharides are thought to promote bowel movements by increasing the number of *Bifidobacterium* and lactic acid bacteria in the intestines [20]. Among the oligosaccharides, isomalto-oligosaccharides function as a growth factor for *Bifidobacterium* [21]. It has been reported that the intake of isomalto-oligosaccharides is effective in increasing *Bifidobacterium* and improving defecation [17,21,22]. A culture study of the gut microbiota of patients with SMID, on long-term enteral nutrition, reported that *Lactobacillus* and *Clostridium perfringens* were not detected, and their gut microbiota had a distinctive composition compared to healthy individuals of the same age [7]. This study also reported that the group receiving enteral nutrition containing oligosaccharides (galactooligosaccharides and lactulose) had significantly higher levels of *Bifidobacterium* than the group receiving enteral nutrition without oligosaccharides. In addition, carnitine supplementation [22] and consumption of kefir fermented milk [8,10] have been reported to reduce constipation symptoms in patients with SMID. However, to the best of our knowledge, there are no reports on constipation in patients with SMID pertaining to the gut microbiota. Moreover, there are few studies on the gut microbiota of patients with SMID using next-generation sequencing analysis. Therefore, the purpose of this study was to investigate changes in constipation symptoms and gut microbiota associated with the intake of malted rice amazake, for six weeks, in patients with SMID.

## 2. Materials and Methods

### 2.1. Study Design and Participants

This was a pilot study on the effect of consumption of malted rice amazake for six weeks in patients with SMID. A control group without malted rice amazake intake or a placebo group was not established due to operational issues of the research facility. Consent was obtained from 14 patients receiving care at the Kishu Rehabilitation Visiting Care Station, Wakayama, Japan. The inclusion criteria were as follows: (1) receiving medical subsidies for SMID, based on diagnosis by a physician, (2) Oshima’s classification [23] 1–9, which indicates bedridden or sitting with support, gait disturbance, and an IQ less than 50. Patients consuming antibiotics within one month before the intervention, other than those on low-dose macrolide therapy, were excluded. Ten patients (five men and five women) were included in this analysis. The protocol for this study was approved by the Ethics Committee of Okayama Prefectural University (Approval No. 17-73) and registered in the clinical trial registration system (UMIN000039406) in accordance with the Declaration of Helsinki.

### 2.2. Malted Rice Amazake

In this study, the test food was malted rice amazake in the paste form (Koji Sweet, Marumi Koji Honten Co., Ltd., Okayama, Japan), which has already been shown to be effective in humans [15,17]. The nutritional components per daily dose (35 g) are shown in Table 1. In accordance with a previous study [16,17], 35 g of the test food was diluted to 150 mL, with water or lukewarm water, and consumed daily for six weeks. A portion of the water volume consumed before the intervention was replaced with 150 mL of water containing 35 g of malted rice amazake. The same test food was infused into the participants by tube feeding, using a tube or syringe.

### 2.3. Investigation

Physical status, the brief-type self-administered diet history questionnaire (BDHQ) [24], and prescription drugs were administered before and after the intervention. The Japanese version of the Constipation Assessment Scale (CAS) [25] was used to investigate the defecation status before and after the intervention. In this study, caregivers responded to the CAS instead of the patients who had difficulty in self-assessment; thus, four out of 10 patients being analyzed were not able to assess all the items of the CAS. Therefore, the evaluation standard of this study was to calculate the percentage of evaluation points, according to the number of items for each patient, and to evaluate the presence of constipation when the percentage exceeded 31.3%, which corresponds to the evaluation standard (five points out of 16) adopted in a previous study [25]. This method of evaluation using the percentage of CAS points is also used in a report for home-care patients with disabilities, including SMID, by Inoue et al. [26].

### 2.4. Fecal Sample Collection

Feces was collected twice, before and after the intervention, by dropping it on the collection sheet and placing it in the stool collection case with a disposable spoon, or by placing feces, excreted on a diaper, in the stool collection case with a disposable spoon. The amount of feces to be collected was approximately 1/2 tsp, and after collection, it was stored at −20 °C.

### 2.5. DNA Extraction

DNA was extracted from frozen feces using an automated nucleic acid extractor (Kurabo Industries Ltd., Osaka, Japan), with some modifications to a previously reported protocol [27]. Frozen stool samples, the size of a grain of rice, were homogenized with 500 µL of lysis buffer (No. 10, Kurabo Industries Ltd., Osaka, Japan) and 0.5 g of 0.1 mm glass beads in a 2 mL vial. The mixture was mechanically disrupted at 4,260 rpm for 50 s at room temperature (20–25 °C), using a tabletop cell disruptor, Cell Destroyer PS 1000 (Bio Medical Science, Tokyo, Japan). The cells were then centrifuged at 12,000× *g* for 5 min at room temperature (20–25 °C). The supernatant (200 µL) was collected and mixed with 150 µL of lysis buffer and 150 µL of proteinase K buffer containing 0.4 mg/mL of proteinase K (No. 2, Kurabo Industries Ltd., Osaka, Japan). DNA was extracted using an automated nucleic acid extractor Gene Prep Star PI-80X (Kurabo Industries Ltd., Osaka, Japan), and DNA concentration was measured using a NanoDrop spectrophotometer ND-1000 (Thermo Fisher Scientific Inc., Wilmington, DE, USA). The extracted DNA samples were stored at −30 °C until use.

### 2.6. 16S rRNA Sequencing

For the analysis of gut microbiota in feces, metagenomic analysis using 16S rRNA sequencing was performed, as previously described [27]. The V3-V4 region of the 16S rRNA gene was amplified by PCR using published primers [28] from DNA samples extracted from feces. The PCR conditions were 95 °C for 3 min, followed by 25 cycles of 95 °C for 30 s, 55 °C for 30 s, 68 °C for 1 min, and a final extension at 68 °C for 5 min. The final extension was performed at 68 °C for 5 min. The resulting PCR products were purified using Agencourt AMPure XP (Beckman Coulter, Inc., Brea, CA, USA), according to the manufacturer’s protocol, and eluted in 50 µL of 10 mM Tris-HCL (pH 8.5). DNA libraries were then prepared using Illumina MiSeq Nextera kit set A (Illumina Inc., San Diego, CA, USA) and sequenced using Illumina MiSeq (Illumina).

### 2.7. Bioinformatics Analysis

FASTQ files (files containing sequences and quality scores), obtained by Illumina pair-end amplicon sequencing of 16S rRNA genes, were processed according to previously reported methods [29]. Processed sequence data were analyzed using the QIIME version 1.9.1 pipeline [30]. Operational taxonomic units (OTUs) were generated using USEARCH [31], based on clusters with 97% similarity, in the SILVA 128 database [32]. OTUs were analyzed from phylum to genus using the SILVA 128 database [32]. In all samples, 10,000 randomly selected reads were used for the analysis.

### 2.8. Statistical Analysis

The data were exported as BIOM files and imported into R (version 3.5.1) for statistical analysis. Alpha diversity was calculated using the *estimate_richness* function of the “phyloseq” R-package based on OTU-level information [33]. In addition, to investigate the effect of malted rice amazake intake on the gut microbiota based on the characteristics of the original gut microbiota, the patients being analyzed were grouped by hierarchical clustering [34] according to the similarity of the gut microbial structure before the intervention. Hierarchical clustering was performed, using the ward.D2 method, by calculating the distance between samples using the Bray–Curtis distance, based on genus-level information. The classification was performed using the rect.hclust function of the “stats” R-package. In this study, analyses were conducted for the overall and for groups using hierarchical clustering. Wilcoxon’s signed rank test and Pearson’s chi-square test were used to compare conditions before and after the intervention, and Mann–Whitney’s *U* test, Kruskal–Wallis test, and Pearson’s chi-square test were used to compare between groups. The Statistics Premium Grad Pack Version 26 (IBM, Armonk, NY, USA) was used for statistical analysis. Measurements were expressed as mean and standard error, and the significance level was set at less than 5% by a two-tailed test.

## 3. Results

### 3.1. Characteristics of the Patients Being Analyzed

The characteristics of the patients are presented in Table 2. Body mass index (BMI) was underweight (BMI: <18.5) in all but one patient. In the five patients under 18 years old, height for age (H/A) showed severe undernutrition (H/A: <85) in four and moderate undernutrition (H/A: 85–90) in one; weight for height (W/H) was severe undernutrition (W/H: <70) in two, moderate undernutrition (W/H: 70–80) in one, mild undernutrition (W/H: 80–90) in two, and normal (W/H: 90–110) in one. Regarding motor function, seven patients were bedridden, one could sit with support, and two had gait disturbance. Regarding nutritional intake methods, three patients were on oral intake, two were on nasal feeding, four were on gastrostomy, and one was on combined oral and nasal feeding. Five patients had no dietary fiber intake at all (0 g), and two had no weaning experience.

### 3.2. Grouping of the Patients Being Analyzed

**Figure 1:**
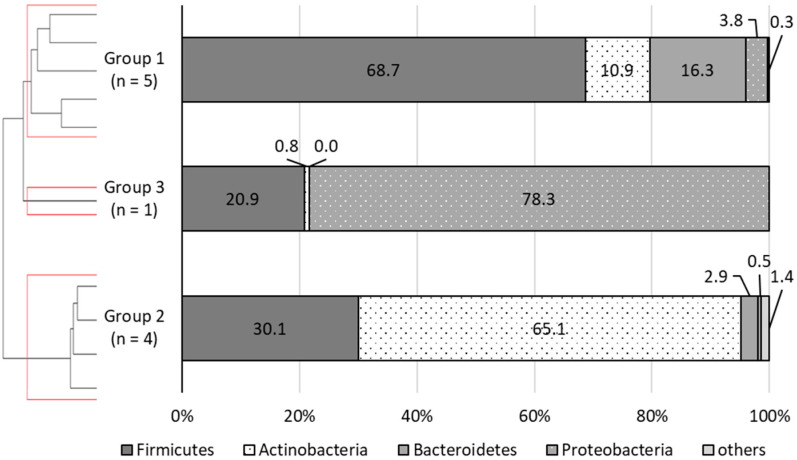
Major four phyla of gut microbiota of each group. The patients being analyzed were classified into three groups by hierarchical clustering. According to the Kruskal–Wallis test, Group 1 had a higher abundance of Firmicutes, whereas Group 2 had a higher abundance of Actinobacteria. Group 3 had a high abundance of Proteobacteria, which did not apply to the other groups.

To investigate the effect of malted rice amazake intake on the gut microbiota, based on the characteristics of the original gut microbiota, the patients were classified into three groups by hierarchical clustering based on their fecal bacterial compositions (Figure 1). According to the Kruskal–Wallis test, Group 1 (*n* = 5) had a higher relative bacterial abundance of Firmicutes (Group 1: 68.7 ± 5.3%, Group 2: 30.1 ± 2.8%, Group 3: 20.9%, *p* = 0.025, Group 1 vs. 2: *p* = 0.025, Group 1 vs. 3: *p* = 0.035), whereas Group 2 (*n* = 4) had a higher abundance of Actinobacteria (Group 1: 10.9 ± 3.9%, Group 2: 65.1 ± 4.7%, Group 3: 0.8%, *p* = 0.025, Group 1 vs. 2: *p* = 0.027, Group 2 vs. 3: *p* = 0.027). Group 3 (*n* = 1) showed a high abundance of Proteobacteria and did not apply to the other groups. The characteristics of each group, except for Group 3, are shown in Table 3. Regarding motor function, in Group 1, two patients were bedridden, and three were sitting with support and gait disturbance, while in Group 2, all four patients were bedridden (*p* = 0.058). About nutritional intake methods, in Group 1, three patients were orally fed, and two were nasally fed; in Group 2, one patient was nasally fed, and three were on gastrostomy, which was significantly different between groups (*p* = 0.043). In terms of experience with weaning food, all five patients in Group 1 had experience, whereas two of the four patients in Group 2 had experience (*p* = 0.073). In other words, all four patients in Group 2 were bedridden and tube fed, and two of them had no experience with weaning food.

### 3.3. Changes Due to the Intervention and Comparison between Groups

#### 3.3.1. Weight, Nutrient Intake, and Prescription Drugs

Changes in body weight and nutrient intake (Table S1), as well as changes in the types of prescription drugs (Table 4), overall and in each group, are shown. There were no significant differences before and after the intervention (*p* > 0.05). In the comparison between groups of nutrient intake, vitamin B_1_ (thiamine), niacin, vitamin B_6_, pantothenic acid, and vitamin C were significantly higher in Group 2 (*p* = 0.016). No one in Group 1 used muscle relaxants, while all in Group 2 used muscle relaxants. The number of muscle relaxants was significantly higher in Group 2 than in Group 1 (*p* = 0.016).

#### 3.3.2. CAS

**Figure 2:**
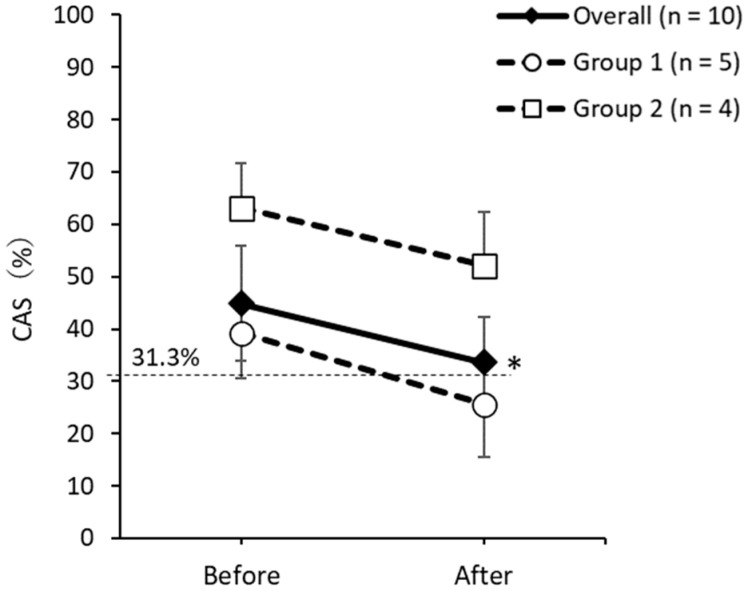
Changes in CAS overall and in each group. CAS, constipation assessment scale. The percentage of people with constipation is ≥ 31.3%. Data are shown as the mean ± SEM. Changes before and after the intervention: Wilcoxon signed rank test. Comparison between groups before and after intervention: Mann–Whitney *U* test. * *p* < 0.05.

The changes in CAS, overall and in each group, are shown in Figure 2. Overall, the CAS was significantly decreased by 44.9 ± 11.0% before and 33.7 ± 8.6% after the intervention (*p* = 0.043), and constipation symptoms were reduced. In Group 1, it was 39.3 ± 16.4% before and 25.6 ± 10.3% after the intervention (*p* = 0.109), and in Group 2, it was 63.1 ± 8.6% before and 52.2 ± 10.1% after the intervention (*p* = 0.180), but there was no significant change within the groups and no significant differences between the groups before (*p* = 0.556) and after (*p* = 0.286). However, both groups showed a decrease in CAS after the intervention, which was similar to the change in overall CAS. The CAS, before and after the intervention, was higher in Group 2 than in Group 1, indicating that Group 2 had more constipation symptoms than Group 1.

#### 3.3.3. Gut Microbiota

**Figure 3:**
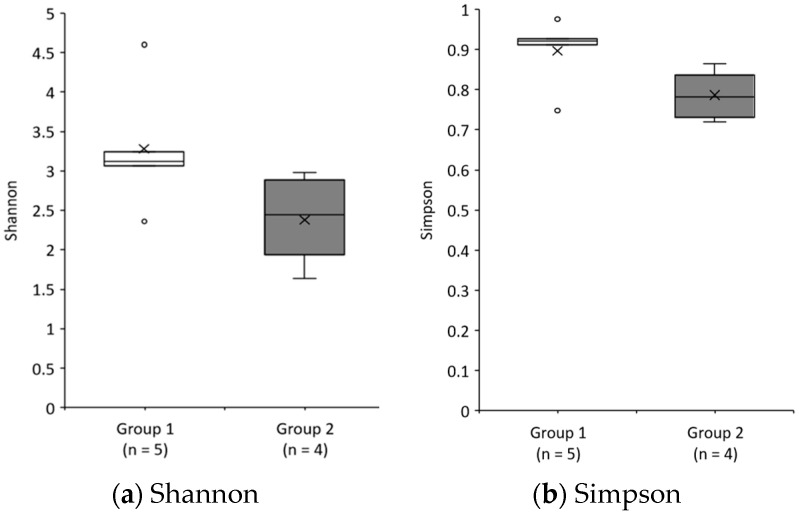
Comparison of gut microbiota diversity between groups before intervention. (**a**) Shannon. (**b**) Simpson. Data are shown as the median (interquartile range). Mann–Whitney *U* test.

Comparisons of the alpha diversity of Shannon and Simpson before the intervention are shown in Figure 3a,b. Shannon and Simpson, in Group 1, were 3.28 ± 0.37 and 0.90 ± 0.04, respectively, and, in Group 2, were 2.38 ± 0.32 and 0.79 ± 0.04, respectively. Although these were not significantly different (*p* = 0.063 and *p* = 0.063, respectively), both Shannon and Simpson indices in Group 1 showed a higher tendency than those in Group 2. There were no significant changes in Shannon (overall: before 2.73 ± 0.29, after 2.52 ± 0.21, *p* = 0.575; Group 1: before 3.28 ± 0.37, after 2.74 ± 0.28, *p* = 0.500; Group 2: before 2.38 ± 0.32, after 2.24 ± 0.41, *p* = 0.144) and Simpson (overall: before 0.81 ± 0.04, after 0.80 ± 0.03, *p* = 0.959; Group 1: before 0.90 ± 0.04, after 0.82 ± 0.05, *p* = 0.345; Group 2: before 0.79 ± 0.04, after 0.77 ± 0.05, *p* = 0.715), overall and in each group, before and after the intervention.

**Figure 4:**
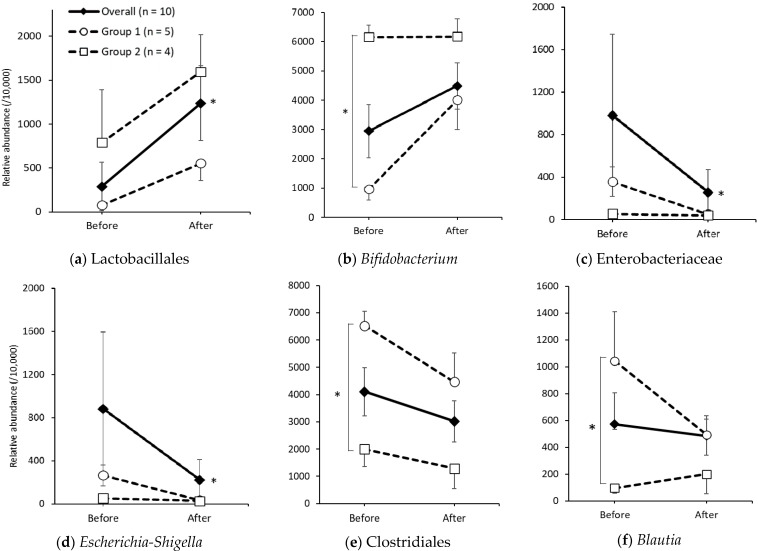
Changes in gut microbiota overall and in each group. (**a**) Lactobacillales. (**b**) *Bifidobacterium*. (**c**) Enterobacteriaceae. (**d**) *Escherichia-Shigella*. (**e**) Clostridiales. (**f**) *Blautia*. The graphs show the gut microbiota, from the order to the genus, that showed significant changes before and after intervention and those that had a significant difference between the groups before intervention. Data are shown as the mean ± SEM. Changes before and after the intervention: Wilcoxon signed rank test. Comparison between groups before and after intervention: Mann–Whitney *U* test. * *p* < 0.05.

At the phylum level, Firmicutes, Actinobacteria, and Bacteroidetes did not change significantly before and after the intervention; however, Proteobacteria showed a significant decrease, overall (*p* = 0.022) and in Group 1 (*p* = 0.043), after the intervention (Table S2). Figure 4a–f show the gut microbiota, from order to genus, of those that had significant changes, before and after the intervention, or those that showed significant differences between groups before the intervention. The Lactobacillales order, classified as many lactate-producing bacteria, was significantly increased (*p* = 0.022) (Figure 4a). There were no significant changes in Lactobacillales in Group 1 (*p* = 0.138) and Group 2 (*p* = 0.144), but both groups showed an increasing change similar to the overall results. For the *Bifidobacterium* genus (Figure 4b), Group 2 had a significantly higher abundance than Group 1 before the intervention (*p* = 0.016). Group 2 showed no change in *Bifidobacterium* (*p* = 0.715) and maintained a higher abundance after the intervention. On the other hand, although there were no significant changes in *Bifidobacterium* overall (*p* = 0.093) and in Group 1 (*p* = 0.080), these changes were increased. Figure 4c,d show changes in the Enterobacteriaceae family belonging to Proteobacteria and *Escherichia-Shigella* at the genus level. Both bacteria showed similar changes, with a significant decrease in Enterobacteriaceae and *Escherichia-Shigella* (*p* = 0.047 and *p* = 0.028, respectively). Enterobacteriaceae and *Escherichia-Shigella*, in Group 2, were low in abundance and did not change after the intervention (*p* = 0.854 and *p* = 0.465, respectively). In Group 1, although there were no significant changes in both bacteria (Enterobacteriaceae: *p* = 0.080, *Escherichia-Shigella*: *p* = 0.080), these showed decreasing changes that were similar overall. The Clostridiales order belonging to Firmicutes is a classification of many butyrate-producing bacteria. Before the intervention, Group 1 had a significantly higher abundance of Clostridiales than Group 2 (*p* = 0.016) (Figure 4e), but there were no changes, overall (*p* = 0.169) and in both groups (Group 1: *p* = 0.138; Group 2: *p* = 0.273), before and after the intervention. The *Blautia* genus, belonging to Firmicutes, an acetic acid-producing bacteria, was significantly higher abundant in Group 1 than in Group 2 (*p* = 0.016) (Figure 4f), but there were no changes overall (*p* = 0.953) and in both groups (Group 1: *p* = 0.345; Group 2: *p* = 0.593) before and after the intervention. Incidentally, there was no correlation with CAS for the bacteria (Figure 4a–f).

## 4. Discussion

The purpose of this study was to investigate the changes in constipation symptoms and gut microbiota, associated with the intake of malted rice amazake for six weeks, in patients with SMID. The results showed a significant decrease in CAS, and at the same time, changes in gut microbiota were observed, as shown by a significant decrease in Proteobacteria phylum, a significant increase in the Lactobacillales order, and a significant decrease in the Enterobacteriaceae family and the *Escherichia-Shigella* genus. In addition, by focusing on the gut microbiota of patients with SMID, and analyzing each group according to the similarity of their gut microbiota before the intervention, we examined the different effects of consuming malted rice amazake based on the characteristics of the original gut microbiota. The results showed that CAS decreased in both Groups 1 and 2, in which Firmicutes and Actinobacteria were high, respectively. As for the changes in the gut microbiota after the intervention, Group 1 showed a significant decrease in Proteobacteria, while Group 2 showed no significant changes in the gut microbiota. *Bifidobacterium*, which has been reported to increase with the intake of isomalto-oligosaccharides, showed an increasing tendency overall and in Group 1. However, the level of *Bifidobacterium* in Group 2 was originally higher, indicating that there were differences in the changes in the gut microbiota between the groups. The results of this study were limited by the small sample size and lack of a control group. However, this is the first study to show that six weeks of malted rice amazake consumption may reduce constipation symptoms in patients with SMID, accompanied by changes in the gut microbiota at the same time. This study also reports a novel finding that shows the possibility of predicting the change in gut microbiota, caused by the intake of malted rice amazake, based on the characteristics of the gut microbiota of patients with SMID.

For constipation symptoms, the overall CAS showed a significant decrease after the intervention compared to before the intervention. In terms of changes before and after the intervention in each group, there were no significant changes in the CAS. This may have been due to the small sample size of each group, which prevented the difference from reaching statistical significance. This result is similar to that of a previous study that examined the effect of amazake consumption on constipation. In a randomized controlled trial in adult women [35], 30 days of amazake consumption increased the frequency and volume of bowel movements and decreased the use of laxatives. A controlled study in female students [16] showed improvement in CAS after 2 weeks of malted rice amazake consumption. Our pilot study of disabled patients at home, who used a home nursing station, including patients with SMID, also showed that six weeks of malted rice amazake consumption improved the CAS [26]. In addition, Yamada et al. [15] reported that, after two weeks of consuming millet amazake, spontaneous defecation was observed in elderly people who could maintain a sitting position and apply abdominal pressure during defecation, and it was effective in improving constipation. These findings consistently indicate that continuous intake of amazake may contribute to the improvement of constipation.

Factors that affect the defecation status of patients with SMID include skeletal deformities in underlying diseases, excessive muscle tone, and impaired intestinal peristalsis [7], especially those caused by central nervous system disorders, which are considered to be a major risk for constipation [36]. The severity of the central nervous system symptoms in Group 2 was higher than that in Group 1, based on motor function, nutrient intake, weaning experience, and medication use, suggesting that the central nervous system may be more severely impaired. The CAS was higher in Group 2. However, in a previous study to improve constipation in patients with SMID, a double-blind comparative study of kefir fermented milk intake for three months by Ino et al. [8] and for 12 weeks by Maki et al. [10] showed an increase in the frequency of bowel movements and a decrease in enemas for some patients with SMID. A retrospective study by Murata et al. [22] pointed out carnitine deficiency in patients with SMID, and showed that carnitine supplementation increased the frequency of defecation and improved constipation by changing the shape of stools. These findings indicate that, even if the major risk of constipation in patients with SMID is due to the damage to the central nervous system, additional diet and nutrients may still be effective in improving constipation. It has also been reported that increasing the intake of dietary fiber and water is an additional strategy to improve constipation in children with neurological disorders [37]. In the present study, the intake of 0.6 g of dietary fiber per daily dose of malted rice amazake and the increased intake of malted rice amazake itself, diluted to 150 mL with water or lukewarm water, may have affected the reduction in constipation symptoms. In other words, the consumption of malted rice amazake for six weeks may contribute to the reduction in constipation symptoms in patients with SMID, with different causative diseases, severity, and gut microbiota. This study also assessed prescribed drugs and nutrient intake, other than malted rice amazake, to validate the changes associated with malted rice amazake consumption as much as possible, and it showed that there were no significant changes during the intervention period. Therefore, the changes in the CAS, obtained in this study, were independent of medication and nutrient intake.

The intake of isomalto-oligosaccharide, contained in malted rice amazake, has been reported to improve defecation and significantly increase *Lactobacillus* and *Bifidobacterium* [20,38]. In this study, the number of *Lactobacillus* was low, and no significant increase or decrease was observed; however, a significant increase was observed in the Lactobacillales order, which is classified as a large number of lactic acid-producing bacteria (*p* = 0.022). No significant changes were observed between the groups (Group 1, *p* = 0.138; Group 2, *p* = 0.144), but there was an increase in Lactobacillales in both groups as well as overall (Figure 3a). *Bifidobacterium* showed an increasing tendency overall (*p* = 0.093) and in Group 1 (*p* = 0.080) (Figure 3b).

Oligosaccharides, such as isomalto-oligosaccharides, are indigestible components, and when they reach the colon, they are utilized by bacteria belonging to Lactobacillales and *Bifidobacterium* in the intestine to promote their growth [39]. It has been reported that lactic acid and acetic acid, one of the short-chain fatty acids (SCFA), which are produced by these bacteria, can promote intestinal peristalsis and prevent constipation, lowering intestinal pH in human studies [20,40] In experiments involving mice, it has been reported that 5-hydroxytryptamine (5-HT) is released from enterochromaffin cells (EC cells) in response to SCFA and stimulates 5-HT_3_ receptors in sensory fibers of the vagus nerve. This sensory information is transmitted to the centrifugal nerve of the vagus nerve, and acetylcholine (ACh) is released from the nerve plexus of the colonic muscular layer, which causes muscle contraction, or intestinal peristalsis [41]. In a previous intervention study using isomalto-oligosaccharide (10 g/day) in constipated patients, fluorescence in situ hybridization (FISH) showed that *Bifidobacteria*, *Lactobacilli*, and *Bacteroides* significantly increased, *Clostridium* significantly decreased, and stool volume and frequency increased [21]. In an intervention study, in which healthy people consumed 10 g or 15 g of isomalto-oligosaccharides daily, the culture methods showed a significant increase in the number and occupancy of *Bifidobacterium*, a significant increase in the number of *Lactobacillus*, a significant decrease in the occupancy of Bacteroidaceae, and a decrease in the number of *Clostridium*, as well as a significant decrease in fecal pH, a decreasing tendency in putrefactive products, and an increase in the frequency of defecation in constipated individuals [20]. In an intervention study of malted rice amazake containing isomalto-oligosaccharides, in middle-aged and elderly people, RT-PCR showed that *Bifidobacterium* and *Akkermansia* were significantly increased in the constipation group, indicating a significant improvement in constipation symptoms [17]. These findings indicate that isomalto-oligosaccharide intake may be effective in improving defecation status as well as increasing the population of *Lactobacillus* (a genus of bacteria in the order Lactobacillales) and *Bifidobacterium*.

In this study, the increase in Lactobacillales overall and in both groups, and the increase in *Bifidobacterium*, overall and in Group 1, after six weeks of malted rice amazake consumption agree with the findings of previous studies, although the intake of isomalto-oligosaccharides was as low as 0.96 g per day (35 g) of malted rice amazake. Therefore, we suggest that these changes in Lactobacillales and *Bifidobacterium* may contribute to the reduction in constipation symptoms overall and in Group 1, and that the mechanism of promoting intestinal peristalsis with the increase in SCFA may have been involved in it. In contrast, in Group 2, there was an increase in Lactobacillales, but no significant change was observed in *Bifidobacterium*, which was already high before the intervention. This suggests that changes in Lactobacillales may contribute to the possible reduction in constipation symptoms in Group 2, but the possible contribution of *Bifidobacterium* cannot be determined.

In the Proteobacteria phylum, there was a significant decrease overall and in Group 1 (Table S2). In Enterobacteriaceae at its family level, a significant decrease overall (*p* = 0.047), and a decreasing tendency in Group 1 (*p* = 0.080), was observed (Figure 4c), and in *Escherichia-Shigella* at its genus level, a significant decrease overall (*p* = 0.028) and a decreasing tendency in Group 1 (*p* = 0.080) was seen (Figure 4d). Enterobacteriaceae have been reported to weaken the intestinal wall and increase inflammatory responses [42]. In a cross-sectional study examining the intestinal microbiota and inflammatory status of three groups (i.e., healthy individuals, ulcerative colitis, and Crohn’s disease), next-generation sequencing showed a significant increase in Enterobacteriaceae, particularly *Escherichia-Shigella*, only in Crohn’s disease patients [43]. In a cross-sectional study of gastrointestinal symptoms in children and adults with Rett syndrome (RTT), based on the gut microbiota and inflammatory status of healthy children, next-generation sequencing showed that RTT develops mild intestinal inflammation, with a concomitant increase in *Escherichia-Shigella* [44].

The species level of *Escherichia-Shigella* includes *Escherichia coli* (*E. coli*), which are partially pathogenic, but mostly harmless, and have been reported to be beneficial for vitamin K₂ production [45] and prevention of colony formation by pathogenic bacteria [46,47]. On the other hand, the *Shigella* species are bacteria that cause bacterial dysentery. In this study, the species of *Escherichia-Shigella* were not classified by bioinformatics analysis, so it is unclear whether the decreasing tendency of *Escherichia-Shigella*, after six weeks of malted rice amazake consumption, is due to pathogenic or harmless species. Therefore, the presence or absence of an inflammatory state in the intestinal tract, due to high levels of Enterobacteriaceae and *Escherichia-Shigella* before the intervention, and their decrease after the intervention should be investigated in the future, using inflammatory indices such as C-reactive protein (CRP), serum IgA, erythrocyte sedimentation rate (ESR), in addition to the analysis of the species of *Escherichia-Shigella*.

When changes in the Clostridiales order, in which butyrate-producing bacteria are often classified, were examined, there were no significant changes overall or in both groups. However, the relative abundance of Clostridiales was significantly higher in Group 1 than in Group 2 before the intervention (*p* = 0.016), and the difference was no longer significant after the intervention (*p* = 0.063) (Figure 4e). Butyrate is an energy source for intestinal epithelial cells, and it influences mucin production, an important function of epithelial cells [48]. Regarding the relationship between butyrate and constipation, Ge et al. [49] reported that gnotobiotic mice transplanted with feces from constipated patients had low levels of butyrate, and that administration of butyric acid to these mice improved fecal frequency and normalized intestinal peristalsis. In addition, while butyrate is used as a pharmaceutical agent to treat chronic constipation [22], Zhu et al. [50] reported that butyrate is more likely to be increased in patients with chronic constipation than in healthy individuals. These findings did not show a consistent association between butyrate levels and constipation.

*Blautia* is an acetic acid-producing bacterium. Acetic acid, as already mentioned, can promote intestinal peristalsis and prevent constipation by lowering intestinal pH in human studies [20,37]. Furuta et al. [51] reported that constipated children with spina bifida have less *Blautia* than healthy children. Kuai et al. [52] also reported that a decreased abundance of *Blautia* was observed in patients with Parkinson’s disease and constipation before the intervention of fecal microbiota transplantation. In this study, Group 2 had significantly less *Blautia* than Group 1 before the intervention, and Group 2 had stronger constipation symptoms than Group 1, which is concordant with the results of previous studies. However, no significant changes in *Blautia* were observed in either group after the consumption of malted rice amazake. Fructo-oligosaccharides (FOS) have been reported to increase the population of *Blautia* [53], but there are no reports on the association between isomalto-oligosaccharides and *Blautia*. Therefore, *Blautia* may have difficulty utilizing the components of malted rice amazake, but further investigation is needed.

In Group 2, constipation symptoms were stronger than those in Group 1, despite higher levels of *Bifidobacterium*. It was observed that the consumption of malted rice amazake for six weeks may reduce constipation symptoms in Group 2; however, there was no significant change in the gut microbiota. Japanese patients have reported age-related changes in the composition of gut microbiota. The reports state that *Bifidobacterium* populations are abundant in preweaning infancy, decrease with increasing age, and the diversity of the gut microbiota increases after weaning [42]. In fact, two patients with SMID in Group 2 had never consumed weaning foods, and Group 2 had a lower diversity of gut microbiota than Group 1. This suggests that the gut microbiota of Group 2 was similar to that of pre-weaned infants. Furthermore, Odamaki et al. also reported that the relative abundance of Clostridia (class level of Clostridiales) was significantly higher in the adult cluster [42]. Moreover, Liu et al. reported that *Blautia* is rarely present in the gut microbiota of infants younger than six months, and it is more prevalent in children older than 12 months [54]. The fact that there were significantly fewer Clostridiales and *Blautia* in Group 2 than in Group 1 suggests that Group 2 is closer to the gut microbiota of infants before weaning. In this study, there was no age difference between Groups 1 and 2. However, analysis from the perspective of age may provide new insights into the unique gut microbiota, such as with Group 2, and this will be a future issue. Another possible cause of constipation in Group 2 was that all the patients in Group 2 were consuming muscle relaxants, while none of the patients in Group 1 were consuming them. Muscle relaxants include tizanidine hydrochloride, dantrolene sodium hydrate, and baclofen. The side effects of each drug listed in the package inserts of prescription drugs include gastrointestinal symptoms, such as constipation, diarrhea, and abdominal distention. The muscle relaxants for treating relatively severe hypertonia in Group 2 may cause more severe symptoms of constipation than those in Group 1.

One patient (No. 5 in Table 2) belonged to Group 3, and the CAS was 0% before and after the intervention, with no constipation symptoms. However, before the intervention, 78.3% of the gut microbiota were Proteobacteria phylum, 78.3% were Enterobacteriaceae family, and 72.8% were *Escherichia-Shigella* genus, but after the intervention, the percentages decreased sharply to 21.7%, 21.6%, and 19.4%, respectively. Lactobacillales and *Bifidobacterium* increased from 1654 to 4509 and from 2 to 175, respectively, before and after the intervention, indicating that the intake of malted rice amazake caused changes in the gut microbiota of Group 3. The patient in Group 3 was the only person consuming antibiotics with low-dose macrolide therapy. Morello et al. reported that infants with vesicoureteral reflux who received continuous low-dose antibiotic prophylaxis (CAP) had higher levels of Enterobacteriaceae and Bacteroidetes than non-CAP infants, and CAP clearly altered the composition of the gut microbiota [55]. This characteristic gut microbiota of Group 3 may be due to the antibiotic’s medication, with low-dose macrolide therapy, in addition to the medical condition of the patient.

Finally, we examined the changes in constipation symptoms and gut microbiota by individual consumption of malted rice amazake. As for the change in constipation symptoms, all 10 patients showed a decrease or maintenance of CAS. In other words, none of the patients experienced worsening of constipation symptoms due to the consumption of malted rice amazake.

This study had several limitations. First, the study did not include a control group. Therefore, whether the changes in the CAS and gut microbiota, obtained by the six-week intervention, were due to the effect of malted rice amazake consumption will require a clear comparative study in the future. In other words, the effect of malted rice amazake consumption on the CAS and gut microbiota can be demonstrated by examining the control group. In this study, constipation symptoms were evaluated using subjective indices. Although the CAS is a validated assessment method, there is a concern that the results obtained in this study were a phenomenon of regression to the mean. In addition, considering that this study was conducted on patients with SMID, who had difficulty in performing their own assessment, future studies should be based on objective constipation indices, such as abdominal ultrasonography. The sample size of this study also limits the generalizability of the results. Of the 10 patients being analyzed in this study, six were judged to have constipation. Although this study has several limitations, it showed that six-week intake of malted rice amazake was associated with a reduction in constipation symptoms in patients with SMID, and it may be effective as a dietary treatment for constipation. In addition, this is the first study to show that constipation symptoms in patients with SMID may be alleviated and may be accompanied by changes in the gut microbiota. Furthermore, the response of gut microbiota to the intervention may differ depending on the composition of the gut microbiota of patients with SMID. In the future, it may become possible to predict changes in constipation symptoms and gut microbiota, associated with the intake of malted rice amazake, based on the characteristics of the gut microbiota among patients with SMID.

## 5. Conclusions

In order to investigate the changes in constipation symptoms and gut microbiota, associated with six weeks of malted rice amazake consumption in patients with SMID, we analyzed overall, and we also examined each group according to the similarity of gut microbiota before the intervention. The results showed that the intake of malted rice amazake for six weeks alleviated the constipation symptoms of the patients with SMID, and at the same time, the gut microbiota changed, but the changes may have been different among the groups.

## Figures and Tables

**Table 1:** The nutritional components of malted rice amazake per daily intake ^1^.

Component	Amount per 35 g/Day
Energy	76.7 kcal
Protein	1.2 g
Fat	0.1 g
Carbohydrate	18.0 g
Sugars	17.4 g
Isomaltose	0.83 g
Panose	0.07 g
Isomaltotriose	0.06 g
Soluble dietary fiber	0.1 g
Insoluble dietary fiber	0.5 g
Sodium	10.1 mg
Water	15.7 g
Ash	0.035 g

^1^ The patients diluted 35 g of malted rice amazake to 150 mL and ingested it daily for six weeks.

**Table 2:** Characteristics of the patients being analyzed.

	^1^ Sex	Age (yr.)	^2^ Ht (m)	^2^ Wt (kg)	^3^ H/A (<18yr.)	^3^ W/H (<18yr.)	^4^ BMI (kg/m^2^)	^5^ Motor Function	^6^ Intake Method	Energy Intake (kcal/d.)	Fiber Intake (g/d.)	Weaning Experience	Diagnosis	^7^ Group
1	F	11	1.19	19.2	82.9	85.3	13.5	Gait disturbance	Oral	1676	8.9	Yes	21st monosomy	1
2	F	11	1.12	15.1	77.8	79.5	12.0	Bedridden	Nasal, oral	2099	18.6	Yes	Leuko- dystrophy	2
3	M	13	1.27	21.0	80.9	80.8	13.0	Bedridden	Oral	642	5.8	Yes	Cerebral palsy	1
4	F	13	1.18	14.6	76.6	66.4	10.5	Bedridden	Gastro	1724	0	No	Cerebral palsy	2
5	M	15	1.28	15.6	76.6	58.9	9.5	Bedridden	Gastro	600	0	Yes	Sequelae of encephalitis	3
6	M	16	1.50	38.0	88.5	91.6	16.9	Bedridden	Nasal	750	0	Yes	Tuberous sclerosis	1
7	F	18	1.29	26.0	-	-	15.6	Bedridden	Gastro	800	0	No	Sequelae of encephalitis	2
8	M	19	1.58	43.0	-	-	17.2	Gait disturbance	Oral	1136	11.9	Yes	Cerebral palsy	1
9	M	20	1.30	25.0	-	-	14.8	Bedridden	Gastro	900	0	Yes	Cerebral palsy	2
10	F	28	1.37	35.0	-	-	18.6	Sit with support	Nasal	900	10.5	Yes	Rett syndrome	1

^1^ M, male; F, female. ^2^ Ht, height; Wt, weight, ^3^ H/A, height for age; W/H, weight for height. H/A and W/H were calculated using the average height and weight of age and gender in the 2000 infant physical growth survey of the Ministry of Health, Labor and Welfare and the 2000 school health statistics survey of the Ministry of Education, Culture, Sports, Science, and Technology. ^4^ BMI, body mass index. ^5^ Motor function is based on Oshima’s classification. ^6^ Oral, oral feeding; Nasal, nasal tube feeding; Gastro, gastronomy. ^7^ Group indicates the number grouped by hierarchical clustering.

**Table 3:** Characteristics of each group before intervention.

	Sex (Male)	Age (yr.)	^1^ Ht (m)	^1^ Wt (kg)	^2^ BMI (kg/m^2^)	Motor Function (*n*)		^3^ Intake Method (*n*)			Weaning Experience (*n*)	
						Bedridden	Sit with Support & Gait Disturbance	Oral	Nasal	Gastro	Yes	No
Group 1 (*n* = 5)	3	17.4 ± 3.0	1.38 ± 0.07	31.2 ± 4.7	15.8 ± 1.1	2	3	3	2	0	5	0
Group 2 (*n* = 4)	1	15.5 ± 2.1	1.22 ± 0.04	20.2 ± 3.1	13.2 ± 1.2	4	0	0	1	3	2	2
*p* value	^a^ 0.294	^b^ 0.905	^b^ 0.190	^b^ 0.190	^b^ 0.190	^a^ 0.058		^a^ 0.043 *			^a^ 0.073	

^1^ Ht, height; Wt, Weight. ^2^ BMI, body mass index. ^3^ Oral, oral feeding; Nasal, nasal tube feeding; Gastro, gastronomy. Data are shown as the mean ± SEM. ^a^ Pearson’s chi-square test. ^b^ Mann–Whitney *U* test. * *p* < 0.05.

**Table 4:** Changes in types of prescription drugs overall and in each group.

Types of Prescription Drugs (Number of Types)	Overall (*n* = 10)		Group 1 (*n* = 5)		Group 2 (*n* = 4)		Group 1 vs. 2 ^b^ *p* Value
	Before	After	Before	After	Before	After	
Laxative	0.6 ± 0.3	0.7 ± 0.3	1.2 ± 0.6	1.4 ± 0.5	0.0 ± 0.0	0.0 ± 0.0	0.190
	^a^ *p* = 0.317		^a^ *p* = 0.317		^a^ *p* = 1.000		
Intestinal peristalsis promoter	0.8 ± 0.4	0.8 ± 0.4	0.2 ± 0.2	0.2 ± 0.2	1.5 ± 0.9	1.5 ± 0.9	0.190
	^a^ *p* = 1.000		^a^ *p* = 1.000		^a^ *p* = 1.000		
Intestinal flora balance improving drug	0.2 ± 0.1	0.2 ± 0.1	0.4 ± 0.2	0.4 ± 0.2	0.0 ± 0.0	0.0 ± 0.0	0.413
	^a^ *p* = 1.000		^a^ *p* = 1.000		^a^ *p* = 1.000		
Anticonvulsant, Antiepileptic drug	2.4 ± 0.7	2.4 ± 0.7	2.2 ± 1.3	2.2 ± 1.3	2.8 ± 0.6	2.8 ± 0.6	0.413
	^a^ *p* = 1.000		^a^ *p* = 1.000		^a^ *p* = 1.000		
Muscle relaxant	1.0 ± 0.5	0.9 ± 0.4	0.0 ± 0.0	0.0 ± 0.0	1.8 ± 0.8	1.8 ± 0.8	0.016 *
	^a^ *p* = 0.317		^a^ *p* = 1.000		^a^ *p* = 1.000		
Gastric acid secretion inhibitor	0.2 ± 0.1	0.1 ± 0.1	0.2 ± 0.2	0.0 ± 0.0	0.3 ± 0.3	0.3 ± 0.3	1.000
	^a^ *p* = 0.317		^a^ *p* = 0.317		^a^ *p* = 1.000		
Antibiotics	0.1 ± 0.1	0.1 ± 0.1	0.0 ± 0.0	0.0 ± 0.0	0.0 ± 0.0	0.0 ± 0.0	1.000
	^a^ *p* = 1.000		^a^ *p* = 1.000		^a^ *p* = 1.000		
[Side effect] Constipation	0.5 ± 0.2	0.5 ± 0.2	0.2 ± 0.2	0.4 ± 0.4	0.8 ± 0.3	0.8 ± 0.3	0.413
	^a^ *p* = 1.000		^a^ *p* = 0.317		^a^ *p* = 1.000		
[Side effect] Diarrhea	0.7±0.2	0.7±0.2	0.2 ± 0.2	0.4 ± 0.2	1.0 ± 0.0	1.0 ± 0.0	0.190
	^a^ *p* = 1.000		^a^ *p* = 0.317		^a^ *p* = 1.000		

Data are shown as the mean ± SEM. ^a^ Wilcoxon signed rank test. ^b^ *p* values indicate a comparison between the groups before intervention. Mann–Whitney *U* test. * *p* < 0.05.

## Data Availability

The dataset used for the analysis of the current study is available from the corresponding author on reasonable request.

## Supplementary Materials

The following are available online at https://www.mdpi.com/article/10.3390/nu13124466/s1, Table S1: Changes in body weight and nutrient intake overall and in each group, Table S2: Changes in gut microbiota overall and in each group.
