# Supplementary material for: Effects of Malted Rice Amazake on Constipation Symptoms and Gut Microbiota in Children and Adults with Severe Motor and Intellectual Disabilities: A Pilot Study
Source: Nutrients. 2021 Dec 14;13(12):4466. doi: 10.3390/nu13124466 (PMC8705246; doi:10.3390/nu13124466)
Supplement: Supplementary file 1 [file nutrients-13-04466-s001.zip › nutrients-1484016-supplementary.pdf]

Supplementary Materials

Table S1. Changes in body weight and nutrient intake overall and in each group [1].

Weight, Nutrients	Overall (*n* = 10)			Group 1 (*n* = 5)			Group 2 (*n* = 4)			Group 1 vs. 2
	Before	After	[a] *p* value	Before	After	[a] *p* value	Before	After	[a] *p* value	[b] *p* value
Weight (kg)	25.3 ± 3.2	25.4 ± 3.1	0.600	31.2 ± 4.7	30.9 ± 4.5	0.593	20.2 ± 3.1	20.9 ± 3.1	0.285	0.190
Energy (kcal)	1123 ± 166	1105 ± 140	1.000	1021 ± 184	1047 ± 156	0.593	1381 ± 317	1305 ± 264	0.317	0.286
Water (ml)	795 ± 122	763 ± 99	0.465	824 ± 174	825 ± 161	1.000	858 ± 215	858 ± 215	0.317	0.730
Protein (g)	43.2 ± 5.3	43.6 ± 4.9	1.000	39.8 ± 6.0	41.7 ± 6.1	0.593	51.6 ± 10.0	50.4 ± 8.9	0.317	0.556
Fat (g)	36.0 ± 6.9	36.0 ± 6.5	1.000	35.1 ± 7.9	35.4 ± 6.5	1.000	42.8 ± 13.8	42.4 ± 13.6	0.317	1.000
Carbohydrate (g)	157 ± 22	151 ± 16	0.465	135 ± 21	137 ± 17	1.000	199 ± 42	181 ± 28	0.317	0.190
Sodium (mg)	1428 ± 394	1481 ± 372	1.000	1719 ± 623	1893 ± 731	0.593	1310 ± 627	1227 ± 545	0.317	0.905
Potassium (mg)	1573 ± 262	1479 ± 202	0.144	1401 ± 211	1344 ± 172	0.285	1974 ± 571	1810 ± 420	0.317	0.556
Calcium (mg)	567 ± 102	563 ± 108	0.715	503 ± 112	481 ± 112	0.285	721 ± 201	740 ± 212	0.317	0.286
Magnesium (mg)	181 ± 23	174 ± 18	0.273	158 ± 16	152 ± 9	0.593	227 ± 45	216 ± 35	0.317	0.286
Phosphorus (mg)	612 ± 100	617 ± 94	0.715	619 ± 99	637 ± 84	0.593	689 ± 214	681 ± 207	0.317	0.905
Iron (mg)	8.5 ± 1.7	8.1 ± 1.7	0.144	7.2 ± 1.7	6.7 ± 1.7	0.285	11.4 ± 3.6	11.0 ± 3.3	0.317	0.730
Zinc (mg)	8.5 ± 1.6	8.5 ± 1.5	1.000	8.1 ± 1.9	8.3 ± 1.8	0.593	10.1 ± 3.2	10.0 ± 3.1	0.317	1.000
Copper (mg)	1.1 ± 0.2	1.1 ± 0.1	0.465	0.9 ± 0.2	0.9 ± 0.2	1.000	1.4 ± 0.3	1.4 ± 0.2	0.317	0.063
Manganese (mg)	2.0 ± 0.5	2.0 ± 0.4	1.000	2.1 ± 0.6	2.2 ± 0.6	0.593	2.1 ± 1.0	2.0 ± 0.9	0.317	0.730
Retinol equivalent (μg)	850 ± 129	795 ± 131	0.144	643 ± 118	554 ± 127	0.285	1150 ± 228	1125 ± 203	0.317	0.111
Vitamin D (μg)	6.4 ± 1.4	6.7 ± 1.4	0.465	5.5 ± 0.8	6.0 ± 0.8	0.593	8.6 ± 3.3	8.7 ± 3.4	0.317	1.000
α-tocopherol (mg)	15.2 ± 4.4	14.9 ± 4.3	0.465	14.3 ± 5.8	14.0 ± 5.9	1.000	19.2 ± 8.6	18.7 ± 8.2	0.317	1.000
Vitamin K (μg)	162 ± 56	121 ± 28	0.273	218 ± 99	140 ± 30	0.285	127 ± 61	123 ± 58	0.317	0.556
Vitamin B_1 (mg)	1.86 ± 0.37	1.84 ± 0.37	0.465	0.84 ± 0.22	0.85 ± 0.21	1.000	3.03 ± 0.26	2.98 ± 0.30	0.317	0.016*
Vitamin B_2 (mg)	1.81 ± 0.27	1.79 ± 0.27	0.465	1.28 ± 0.32	1.23 ± 0.32	0.285	2.56 ± 0.28	2.57 ± 0.29	0.317	0.063
Niacin (mgNE)	15.7 ± 2.4	16.1 ± 2.3	1.000	10.9 ± 1.6	12.1 ± 2.2	0.593	23.7 ± 1.7	23.2 ± 1.3	0.317	0.016*
Vitamin B_6 (mg)	2.14 ± 0.35	2.12 ± 0.33	0.715	1.24 ± 0.31	1.26 ± 0.30	0.593	3.24 ± 0.09	3.17 ± 0.08	0.317	0.016*
Vitamin B_{12} (mg)	3.92 ± 0.58	4.33 ± 0.67	0.068	3.75 ± 0.32	4.52 ± 0.71	0.109	4.62 ± 1.34	4.69 ± 1.41	0.317	1.000
Folic acid (μg)	295 ± 41	273 ± 37	0.144	218 ± 36	187 ± 22	0.285	409 ± 55	392 ± 43	0.317	0.063
Pantothenic acid (mg)	7.3 ± 1.1	7.2 ± 1.1	0.465	4.9 ± 0.8	4.9 ± 0.8	1.000	10.6 ± 1.6	10.4 ± 1.6	0.317	0.016*

Vitamin C (mg)	181 ± 34	173 ± 31	0.273	98 ± 25	96 ± 27	0.593	287 ± 30	270 ± 20	0.317	0.016 *
Soluble dietary fiber (g)	2.8 ± 1.3	2.5 ± 1.3	0.144	3.5 ± 1.8	3.1 ± 1.9	0.285	2.6 ± 2.6	2.3 ± 2.3	0.317	0.286
Insoluble dietary fiber (g)	2.7 ± 1.2	2.1 ± 0.9	0.144	3.8 ± 1.7	3.2 ± 1.3	0.285	2.0 ± 2.0	1.3 ± 1.3	0.317	0.556
Total dietary fiber (g)	5.6 ± 2.1	4.7 ± 1.7	0.144	7.4 ± 2.1	6.5 ± 1.7	0.285	4.6 ± 4.6	3.6 ± 3.6	0.317	0.413
Sodium chloride amount (g)	3.8 ± 0.9	3.9 ± 1.0	1.000	4.7 ± 1.4	5.1 ± 1.7	0.593	3.3 ± 1.6	3.1 ± 1.4	0.317	0.730

[1] Data are shown as the mean ± SEM. [a] Wilcoxon signed rank test. [b] p values indicate a comparison between the groups before intervention. Mann-Whitney U test. * $p < 0.05$.

Table S2. Changes in gut microbiota overall and in each group [1].

Phylum	Class	Order	Family	Genus	Overall (*n* = 10)			Group 1 (*n* = 5)			Group 2 (*n* = 4)			Group 1 vs. 2
					Before	After	[a] *p* value	Before	After	[a] *p* value	Before	After	[a] *p* value	[b] *p* value
**Firmicutes**					4849 ± 698	4608 ± 619	0.878	6871 ± 553	5207 ± 998	0.225	3011 ± 282	3202 ± 391	1.000	0.016 *
	Bacilli				525 ± 277	1381 ± 425	0.022*	85 ± 24	563 ± 202	0.138	794 ± 604	1621 ± 412	0.144	0.063
		Lactoba-cillales			519 ± 277	1365 ± 426	0.022*	76 ± 25	553 ± 198	0.138	789 ± 602	1595 ± 425	0.144	0.063
			Lactoba-cillaceae		250 ± 250	462 ± 299	0.249	0 ± 0	0 ± 0	0.317	624 ± 624	1126 ± 652	0.715	0.286
				Pediococ-cus	213 ± 213	416 ± 282	0.180	0 ± 0	0 ± 0	1.000	531 ± 531	1041 ± 615	0.180	0.556
			Strepto-coccaceae		31 ± 10	357 ± 343	0.515	38 ± 17	18 ± 7	0.500	29 ± 15	10 ± 6	0.285	0.905
				Strepto-coccus	30 ± 10	356 ± 343	0.514	37 ± 17	18 ± 7	0.500	29 ± 15	9 ± 5	0.276	0.905
			Entero-coccacea	*Entero-coccus*	238 ± 160	505 ± 122	0.169	37 ± 22	500 ± 180	0.138	135 ± 65	422 ± 208	0.273	0.111

Clos-tridia				4106 ± 888	3020 ± 753	0.169	6527 ± 520	4469 ± 1046	0.138	2000 ± 642	1290 ± 738	0.273	0.016 *
	Clostrid-iales			4105 ± 888	3020 ± 753	0.169	6526 ± 520	4469 ± 1046	0.138	2000 ± 642	1290 ± 738	0.273	0.016 *
		Lachno-spiraceae		3008 ± 779	2623 ± 647	0.646	4793 ± 987	3870 ± 853	0.345	1434 ± 451	1094 ± 704	0.715	0.063
			Anaerosti-pes	572 ± 485	452 ± 355	0.799	1043 ± 972	799 ± 711	0.500	127 ± 41	130 ± 34	1.000	0.730
			[Eubacte-rium] hallii group	115 ± 64	453 ± 288	0.225	190 ± 118	531 ± 481	0.465	50 ± 49	468 ± 468	0.317	0.905
			Lachno-clostrid-ium	174 ± 64	207 ± 124	0.721	198 ± 102	369 ± 236	0.500	187 ± 104	51 ± 30	0.068	1.000
			[Rumino-coccus] torques group	462 ± 185	309 ± 122	0.477	743 ± 307	561 ± 187	0.893	226 ± 173	49 ± 17	0.285	0.190
			Blautia	573 ± 234	486 ± 151	0.953	1044 ± 368	495 ± 114	0.345	96 ± 38	200 ± 144	0.593	0.016 *
		Rumino-coccaceae		974 ± 326	279 ± 120	0.066	1536 ± 530	433 ± 226	0.225	505 ± 231	127 ± 64	0.109	0.190
			Sub-doligranu-lum	130 ± 123	12 ± 9	0.058	248 ± 248	19 ± 19	0.414	15 ± 8	7 ± 4	0.109	0.413
			[Rumino-coccus]	137 ± 116	191 ± 122	0.686	268 ± 227	382 ± 220	1.000	7 ± 7	0 ± 0	0.317	0.413

				gauvreauii group										
				Feacali-bacterium	263 ± 234	11 ± 8	0.225	484 ± 469	3 ± 2	0.180	53 ± 53	21 ± 20	0.655	0.730
	Erysipe-lotrichia	Erysipe-lotricha-les	Erysipe-lotricha-ceae		126 ± 36	117 ± 31	0.878	180 ± 59	155 ± 39	0.893	87 ± 36	91 ± 55	0.713	0.413
				Erysipela-toclostrid-ium	86 ± 36	76 ± 23	0.721	127 ± 67	100 ± 36	0.893	54 ± 24	58 ± 36	0.713	0.905
	Nega-tivicutes	Seleno-mona-dales			92 ± 37	90 ± 76	0.260	80 ± 41	20 ± 19	0.068	131 ± 78	201 ± 189	1.000	0.556
**Actinobacteria**					3155 ± 907	4907 ± 712	0.093	1088 ± 388	4431 ± 910	0.080	6508 ± 470	6588 ± 445	0.715	0.016 *
	Actino-bacteria				2955 ± 913	4720 ± 783	0.059	960 ± 367	4230 ± 973	0.080	6187 ± 392	6465 ± 446	0.273	0.016 *
		Bifidobac-teriales	Bifidobac-teriaceae	*Bifidobac-terium*	2948 ± 910	4492 ± 788	0.093	958 ± 366	4008 ± 1005	0.080	6172 ± 394	6176 ± 613	0.715	0.016 *
		Coryne-bacte-riales			4 ± 3	176 ± 107	0.063	1 ± 0	203 ± 189	0.144	10 ± 7	187 ± 152	0.285	0.111
			Coryne-bacteri-aceae		4 ± 3	176 ± 107	0.063	0 ± 0	203 ± 189	0.144	10 ± 7	187 ± 152	0.285	0.111
				Coryne-bacterium 1	0 ± 0	172 ± 106	0.115	0 ± 0	198 ± 188	0.285	0 ± 0	183 ± 149	0.285	1.000

	Coriobacteriia	Coriobacteriales	Coriobacteriaceae		201 ± 68	187 ± 43	0.646	128 ± 52	202 ± 72	0.080	321 ± 147	123 ± 32	0.144	0.286
				Collinesella	94 ± 65	89 ± 36	0.753	18 ± 18	83 ± 49	0.273	213 ± 151	39 ± 27	0.180	0.413
**Bacteroidetes**					931 ± 408	132 ± 65	0.051	1633 ± 745	135 ± 80	0.080	287 ± 182	158 ± 151	0.109	0.111
	Bacteroidia	Bacteroidales			931 ± 428	132 ± 68	0.051	1632 ± 745	135 ± 80	0.080	287 ± 182	158 ± 151	0.109	0.111
			Bacteroidaceae	*Bacteroides*	653 ± 377	96 ± 57	0.173	1130 ± 714	80 ± 58	0.225	221 ± 150	138 ± 131	0.109	0.413
			Rikenellaceae	*Alistipes*	197 ± 125	23 ± 12	0.141	369 ± 235	46 ± 21	0.345	31 ± 17	0 ± 0	0.109	0.730
**Proteobacteria**					993 ± 728	262 ± 203	0.022*	378 ± 134	56 ± 25	0.043*	54 ± 43	42 ± 18	0.715	0.063
	Gammaproteobacteria				981 ± 765	261 ± 213	0.053	355 ± 140	55 ± 25	0.080	53 ± 43	41 ± 18	0.854	0.111
		Enterobacteriales	Enterobacteriaceae		981 ± 765	256 ± 212	0.047*	355 ± 140	49 ± 25	0.080	52 ± 43	40 ± 18	0.854	0.111
				Escherichia-Shigella	881 ± 713	222 ± 191	0.028*	265 ± 99	35 ± 23	0.080	52 ± 43	27 ± 15	0.465	0.111

[1] Based on the relative abundance of 10,000 in each intestinal bacterium, changes in the bacterium that has the relative abundance higher than 100 (1%) are shown. Data are shown as the mean ± SEM. [a] Wilcoxon signed rank test. [b] p values indicate a comparison between the groups before intervention. Mann-Whitney U test. * $p < 0.05$.
